# Congratulatory Message

**DOI:** 10.31662/jmaj.s0005

**Published:** 2018-09-28

**Authors:** Howard Bauchner

**Affiliations:** 1 JAMA and the JAMA Network; 2 American Medical Association

Dear Dr. Yokokura:

Congratulations on the launch of JMA Journal by the Japan Medical Association (JMA) and Japanese Association of Medical Sciences (JAMS). As an official English medical journal covering all aspects of medicine and medical care, including original research, health policy, and global health, I am sure it will be a success not only with clinical investigators in Japan, but worldwide. The publication of this type of comprehensive medical journal is the first challenge in Japan. We sincerely hope that the JMA Journal will promote international medical science and contribute to the improvement of the quality of health care by being read and cited by many people.

If I or any of my staff can be of any help in this important endeavor please let us know.

Good luck with this important initiative of the JMA and JAMS.

Sincerely,

Howard Bauchner, MD

Editor in Chief, JAMA and the JAMA Network (2011-)

Senior Vice-President, American Medical Association

